# TREM2 protects against cerebral ischemia/reperfusion injury

**DOI:** 10.1186/s13041-017-0296-9

**Published:** 2017-06-07

**Authors:** Rong Wu, Xiangpen Li, Pengfei Xu, Likui Huang, Jinping Cheng, Xiaolong Huang, Jingru Jiang, Long-Jun Wu, Yamei Tang

**Affiliations:** 1 Department of Neurology, Sun Yat-Sen Memorial Hospital, Sun Yat-Sen University, Number 107, Yan Jiang Xi Road, Guangzhou, Guangdong Province 510120 China; 2 Key Laboratory of Malignant Tumor Gene Regulation and Target Therapy of Guangdong Higher Education Institutes, Sun Yat-Sen University, Guangzhou, 510120 China; 3 Guangdong Province Key Laboratory of Brain Function and Disease, Zhongshan School of Medicine, Sun Yat-Sen University, 74 Zhongshan 2nd Road, Guangzhou, 510080 China; 4 Department of Cell Biology and Neuroscience, Rutgers University, Piscataway, NJ 08854 USA; 5 Department of Neurology, Mayo Clinic, Rochester, MN 55905 USA

**Keywords:** Inflammation, Ischemia/Reperfusion injury, Microglia, TREM2

## Abstract

Although post-ischemic inflammation induced by the innate immune response is considered an essential step in the progression of cerebral ischemia injury, the role of triggering receptor expressed on myeloid cells 2 (TREM2) in the pathogenesis of ischemic stroke remains to be elucidated. Here, we found that the transcriptional and post-transcriptional levels of TREM2 were increased in cultured primary microglia after oxygen-glucose deprivation and reoxygenation and in the ischemic penumbra of the cerebral cortex after middle cerebral artery occlusion (MCAO) and reperfusion in mice. TREM2 was mainly expressed in microglia, but not in astrocytes, neurons, or oligodendrocytes in mice subjected to MCAO. Manipulating TREM2 expression levels in vitro and in vivo significantly regulated the production of pro- and anti-inflammatory mediators after ischemic stroke. TREM2 overexpression markedly suppressed the inflammatory response and neuronal apoptosis. By contrast, TREM2 gene silencing intensified the inflammatory response, increased neuronal apoptosis and infarct volume, and further exacerbated neurological dysfunction. Our study demonstrated that TREM2 protects against cerebral ischemia/reperfusion injury through the aspect of post-ischemic inflammatory response and neuronal apoptosis. Pharmacological targeting of TREM2 to suppress the inflammatory response may provide a new approach for developing therapeutic strategies in the treatment of ischemic stroke and other cerebrovascular diseases.

**Electronic supplementary material:**

The online version of this article (doi:10.1186/s13041-017-0296-9) contains supplementary material, which is available to authorized users.

## Introduction

Stroke is the leading cause of disability and mortality worldwide. Ischemic stroke caused by the sudden occlusion of a blood vessel by a thrombus or embolism accounts for approximately 80% of all stroke cases [1]. Ischemic stroke is a common vascular disease in the central nervous system (CNS) [2]. Acute cerebral ischemia elicits an immune response that leads to a cascade of events culminating in neuronal death and injury to supportive structures in the brain [3]. Timely pharmacological thrombolysis is the most effective treatment at present. However, pharmacological thrombolysis is limited by its strict and narrow therapeutic window (less than 4.5h), complications derived essentially from the risk of hemorrhage, and the potential damage from reperfusion/ischemic injury [4]. Several mechanisms are thought to be involved in the pathogenesis of ischemic stroke, including excitatory toxicity, oxidative stress, inflammation, and apoptosis [5]. Post-ischemic inflammation induced by the immune response is an essential step in the progression of cerebral ischemia injury [6].

Microglia, considered the macrophages of the CNS, are involved in chronic inflammation [7] and are activated early after ischemia, preceding the invasion of blood-borne immune cells [8]. Microglia can produce pro-inflammatory mediators and neurotoxic compounds, such as interleukin (IL)-1β, IL-6, tumor necrosis factor (TNF)-α, reactive oxygen species, nitric oxide, and prostaglandin E2 [9, 10], which are important determinants of neuronal death in cerebral ischemia. Thus, microglial activation is critical for the defense of neural parenchyma against brain ischemia [11]. Ischemic stroke is a powerful stimulus that disables endogenous inhibitory signaling and triggers significant microglial activation [12]. Activated microglia function to either exacerbate ischemic injury or induce repair and regeneration, depending on the different signals received by microglial receptors [13].

Triggering receptor expressed on myeloid cells 2(TREM2), an important innate immune receptor in the brain, is localized mainly on microglia [14], and has been proved to be highly enriched in microglia compared to the whole brain after using Direct RNA Sequencing(DRS) [15]. TREM2 is a single-spanning membrane receptor belonging to the immunoglobulin and lectin-like superfamily [16]. Coupled with the transmembrane signaling adaptor DAP12, TREM2 is involved in a variety of physiological processes, such as pro-inflammatory reactions and phagocytosis of cell debris, as well as with apoptotic neurons and Aβ protein [17–20]. TREM2 suppresses the inflammatory response in vitro by repression of microglia-mediated cytokine production and secretion [21], and the anti-inflammatory effect of TREM2 was shown in an animal model of multiple sclerosis [22]. Loss of function of TREM2 may also contribute to the pathogenesis of Alzheimer’s disease, in which chronic inflammatory responses occur [23, 24]. In addition, TREM2 deficiency attenuates phagocytic activities of microglia in experimental stroke [25]. However, the precise role of TREM2 in ischemic stroke inflammatory response remains elusive. Therefore, in the present study, we investigated the expression of TREM2 and the effects of TREM2 on the production of inflammatory mediators and on neuronal injury in cultured primary microglia after oxygen-glucose deprivation and reoxygenation (OGDR). We also examined ischemic penumbrae in the cerebral cortex of mice after inducing middle cerebral artery occlusion (MCAO) and reperfusion injury.

## Results

### Increased expression of TREM2 after ischemic stroke in vitro and in vivo

**Fig. 1:**
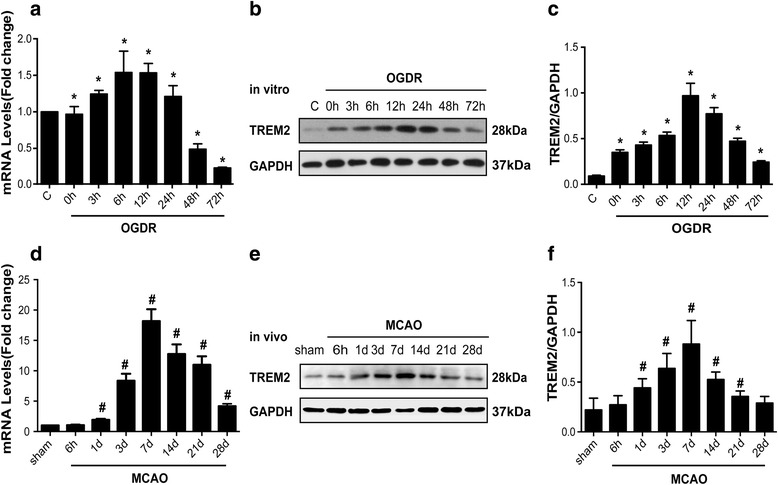
TREM2 is upregulated in OGDR and MCAO ischemic models. TREM2 levels in primary mouse microglia 0, 3, 6, 12, 24, 48, and 72h after OGDR as determined by quantitative real-time PCR (**a**) and western blotting (**b**). **c** Densitometric analysis of TREM2 (relative to GAPDH) from western blotting data in (**b**) presented as mean ± SEM; *n* = 6; *P < 0.05 compared with control. TREM2 levels in mice 6h, 1d, 3d, 7d, 14d, 21d and 28d after MCAO were detected by quantitative real-time PCR (**d**) and western blotting (**e**). **f** Densitometric analysis of TREM2 relative to GAPDH from data in (**e**) presented as mean ± SEM; *n* = 6; ^#^ *P* < 0.05 compared with sham

To investigate the role of TREM2 during ischemic stroke, we first examined cultured primary microglia cells that had been subjected to OGDR. The results of quantitative real-time PCR (Fig. 1a) and western blotting (Fig. 1b and c) analyses indicated that OGDR significantly enhanced microglial TREM2 expression in a time-dependent manner at both transcriptional and post-transcriptional levels, with a peak expression of TREM2 12h after reoxygenation. We then examined the mRNA (Fig. 1d) and protein (Fig. 1e and f) expression levels of TREM2 after reperfusion in mice subjected to MCAO and found that TREM2 mRNA and protein were markedly increased in the ischemic cerebral hemisphere (ischemic core and penumbra), with a peak expression 7 days after the MCAO. These results provided in vitro and in vivo evidence that TREM2 was involved in ischemic stroke.

### Up-regulated TREM2 is localized in microglia in MCAO mice

**Fig. 2:**
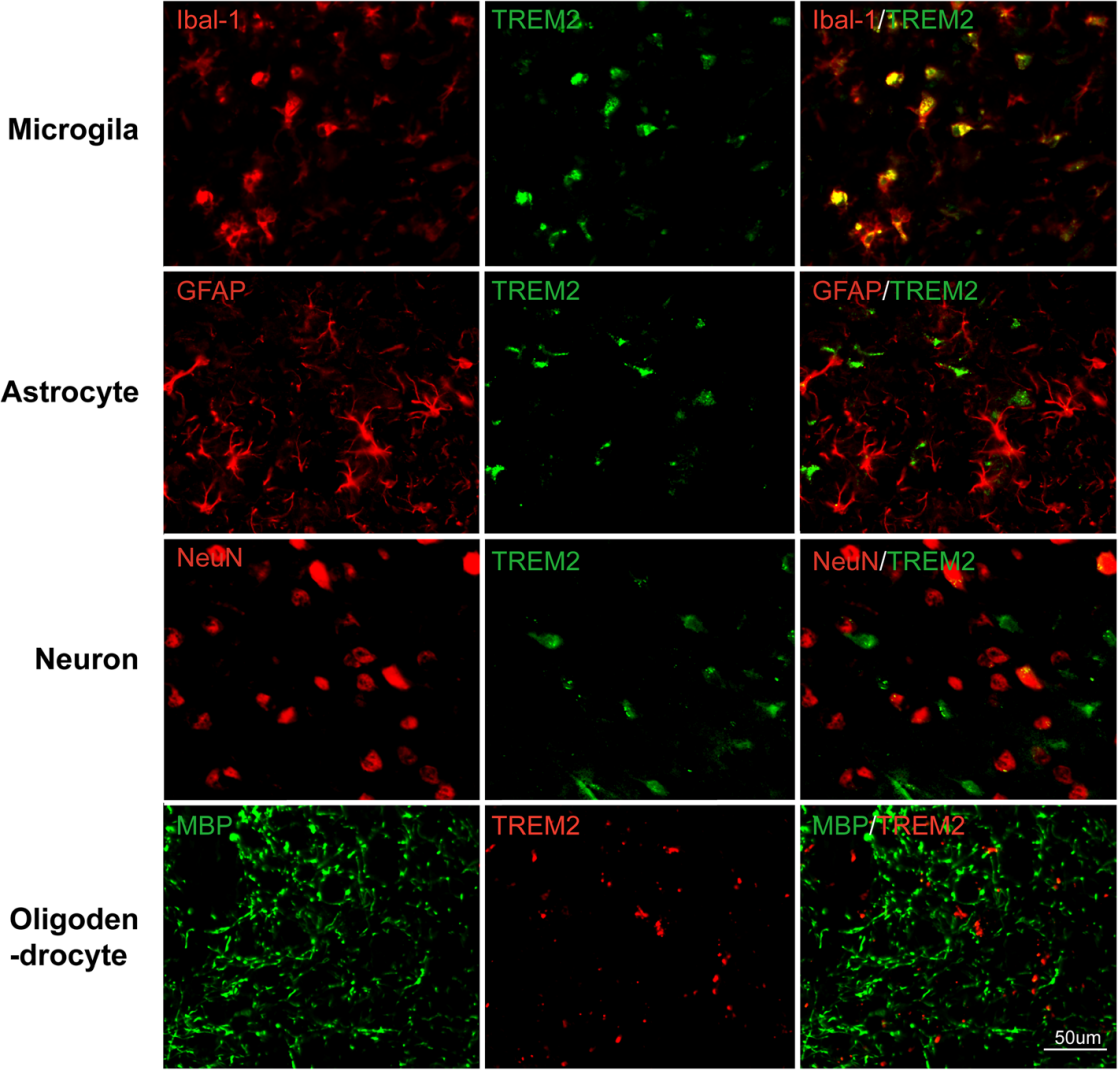
TREM2 colocalizes with Iba1^+^microglia, but not with NeuN^+^ neurons, MBP^+^ myelin, or GFAP^+^ astrocytes in mice subjected to MCAO. Scale bar, 50 μm

We then determined the type of cell responsible for the increased cerebral TREM2 after ischemia. Seven days after MCAO, the ischemic cerebral hemisphere was sliced and subjected to immunohistochemistry. As revealed by the localization of immunofluorescence signals for TREM2 and the microglial maker Iba-1, astrocytic marker GFAP, neuronal marker NeuN, and oligodendrocytic marker myelin basic protein (MBP), we found that TREM2 was mainly expressed in microglia but not in astrocytes, neurons, or myelin in vivo (Fig. 2). Additionally, we have included staining results from normal mouse brain (Additional file 1: Figure 1).

### TREM2 silencing exacerbates stroke outcomes

**Fig. 3:**
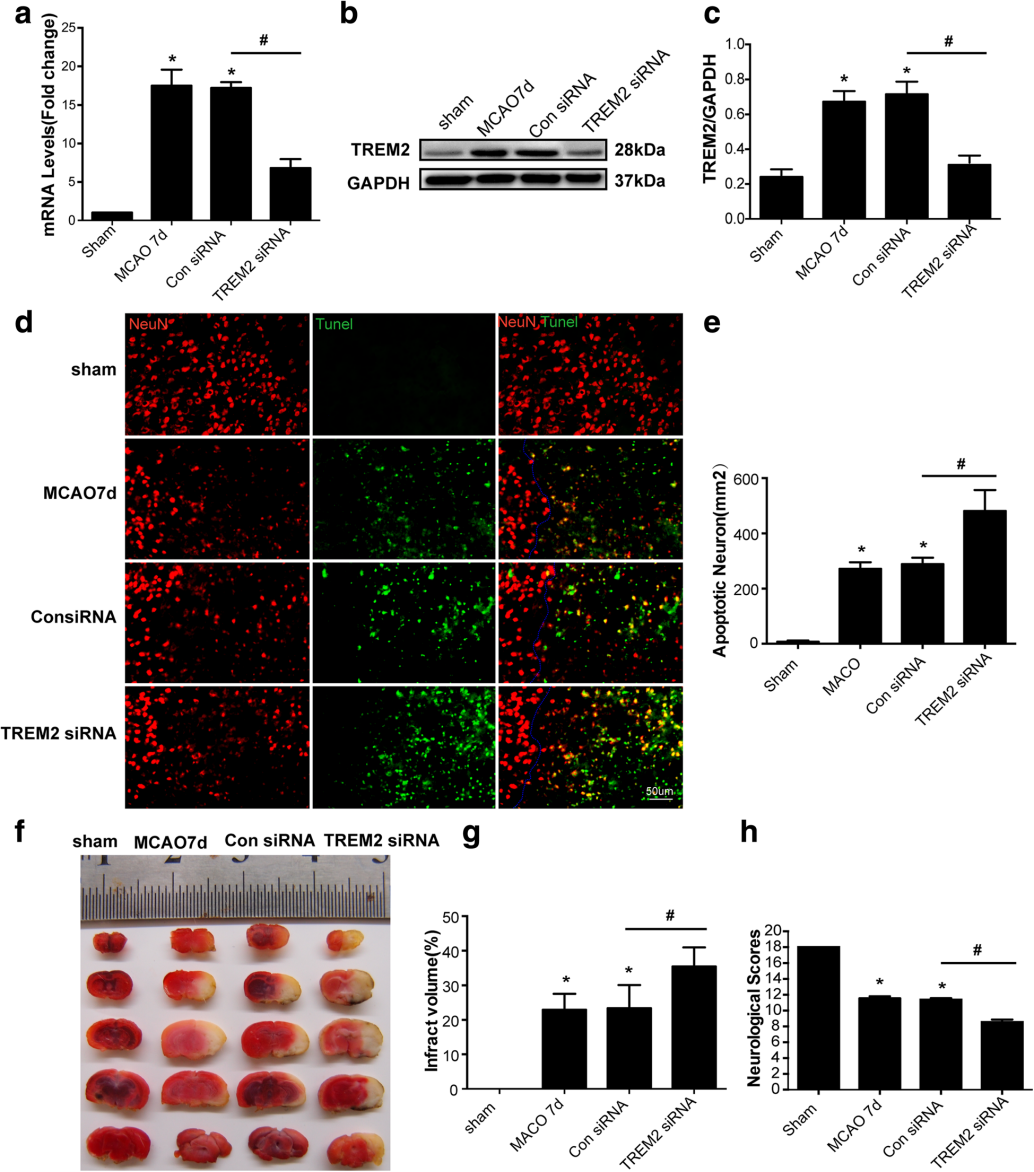
TREM2 silencing exacerbates stroke outcomes. Tissue homogenates from the ischemic cerebral hemisphere (ischemic core and penumbra) of mice that underwent MCAO and 7 days of the indicated treatment were subjected to quantitative real-time PCR (**a**) and western blotting (**b**) and densitometric analysis (**c**) of TREM2 relative to GAPDH proteins.**d** Immunofluorescence labeling with NeuN (*red*) and TUNEL (*green*) of the indicated groups in vivo. **e** The percentage of apoptotic neurons was calculated using image analysis software. Quantified data are shown as mean ± SEM; *n* = 6. Scale bar, 50 μm. **f** Representative samples of TTC-stained brain sections showing the infarcted areas in white. **g** The infarct volume was determined using image analysis and expressed as a percentage of the whole cerebral tissue. Data are presented as mean ± SEM; *n* = 5. **h** Neurological function assessed by the Modified Garcia Score. Data are presented as mean ± SEM; *n* = 16; *P < 0.05 compared with sham; ^#^ *P* < 0.05 compared with the siRNA control. MCAO 7d, 7 days after mice were subjected to MCAO only; Con siRNA, mice subjected to MCAO followed by injections of scrambled siRNA for 7 days; TREM2 siRNA, mice subjected to MCAO followed by TREM2 siRNA injections for 7 days

To confirm the role of TREM2 during ischemic stroke in vivo, we used the TREM2 siRNA approach. Knockdown fragments against TREM2 were designed and synthesized, and the siRNA efficiency was confirmed in vivo. Mice subjected to MCAO were injected with TREM2 siRNA as previously reported [26, 27]. Quantitative RT-PCR and western blotting were used to quantify the amount of TREM2 relative to GAPDH proteins in the ischemic infarction to verify the in vivo knockdown efficiency. After TREM2 siRNA injections for 7 days, the reduced mRNA level (Fig. 3a) and protein expression (Fig. 3b and c) of TREM2 in mice previously subjected to MCAO were significantly suppressed. Next, tissue slices from MCAO mice injected with TREM2 siRNA fragments for 7 days were subjected to immunohistochemistry to determine the percentage of apoptotic neurons. We found significant neuronal apoptosis 7 days after MCAO and reperfusion, and the knockdown of TREM2 following 7 days of TREM2 siRNA injections further exacerbated this neuronal apoptosis (Fig. 3d and e).

We also examined the effects of TREM2 deficiency on ischemic injury in MCAO mice. An analysis of TTC-stained brain sections using imaging software showed that the infarct volume 7 days after reperfusion was significantly larger than that in the sham group (Fig. 3f). Genetic knockdown of TREM2 resulted in a further increase in infarct volume, which was significantly greater than that in the scrambled siRNA-injected group (Fig. 3f and g). The neurological deficit score was determined to evaluate neurological function after MCAO. MCAO markedly reduced neurological function, and mice with the TREM2 knockdown showed the worst neurological dysfunction scores (Fig. 3h). These results indicated that TREM2 was protective in MCAO mice and that inhibition of the up-regulated TREM2 expression following MCAO exacerbated stroke outcomes.

### TREM2 is critical for the inflammatory response following OGDR

**Fig. 4:**
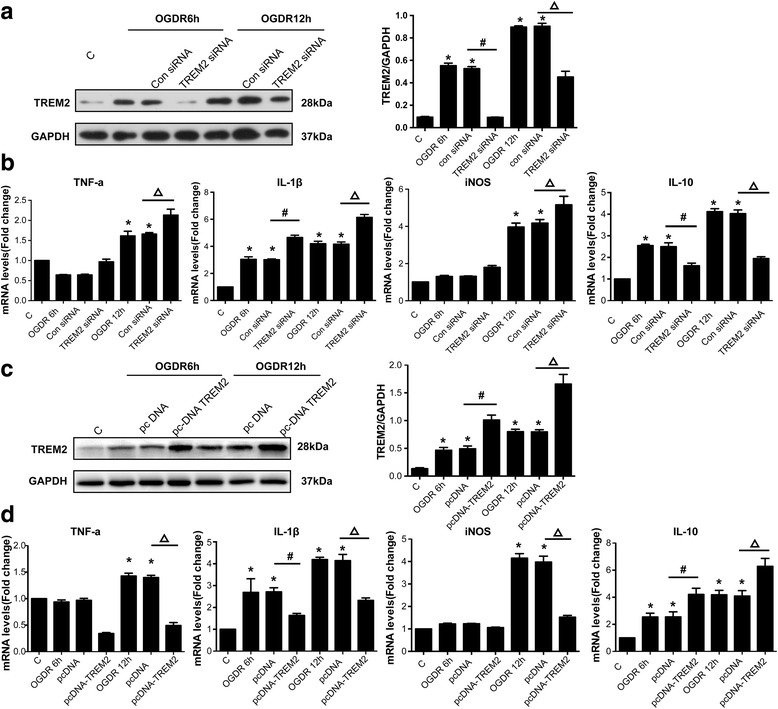
TREM2 is critical for the inflammatory response following OGDR. **a** OGDR was induced in cells, and the cells were transfected 6h and 12h later with TREM2 siRNA. TREM2 and GAPDH protein levels were measured by western blotting. Densitometric analysis of TREM2 relative to GAPDH is shown as mean ± SEM; *n* = 5; *P < 0.05 compared with control group; ^#^ *P* < 0.05 compared with siRNA control in cells transfected 6h after OGDR (OGDR6h + Con siRNA); ^Δ^ *P* < 0.05 compared with siRNA control in cells transfected 12h after OGDR (OGDR12h + Con siRNA). **b** Quantitative real-time PCR was performed to detect the mRNA levels of TNF-α, IL-1β, iNOS, and IL-10 in cells transfected with TREM2 siRNA 6h and 12h after OGDR. Quantified fold changes are shown as mean ± SEM; *n* = 6; *P < 0.05 compared with control group; ^#^ *P* < 0.05 compared with OGDR 6h + Con siRNA group; ^Δ^ *P* < 0.05 compared with OGDR 12h + Con siRNA group. **c** Overexpression of TREM2 6h and 12h after OGDR. TREM2 protein levels were measured by western blotting. Densitometric analysis of TREM2 relative to GAPDH is shown as mean ± SEM; *n* = 5; *P < 0.05 compared with control group; ^#^ *P* < 0.05 compared with pcDNA vector control transfected 6h after OGDR (pcDNA group); ^Δ^ *P* < 0.05 compared with pcDNA vector control transfected 12h after OGDR. **d** Quantitative real-time PCR was performed to detect the mRNA levels of inflammatory factors following TREM2 overexpression 6h and 12h after OGDR. Quantified fold changes are shown as mean ± SEM; *n* = 6; *P < 0.05 compared with control group; ^#^ *P* < 0.05 compared with pcDNA vector control transfected 6h after OGDR group; ^Δ^ *P* < 0.05 compared with pcDNA vector control transfected 12h after OGDR group

Inflammation is a pivotal pathological process during ischemic stroke [2, 28]. Increased levels of IL-1β and TNF-α after ischemia are correlated with infarct severity [29, 30]. IL-10, however, is an anti-inflammatory molecule that can suppress the production of a variety of pro-inflammatory molecules, including TNF-α, IL-1β, and IL-8 [30]. We found that levels of TNF-α, IL-1β, iNOS, and IL-10 were all significantly upregulated 12h after OGDR (Fig. 4b and d). TREM2 is reportedly critical for age-related neuroinflammation and regulation of inflammatory mediators in senescence-accelerated mice [31]. Therefore, we next examined whether TREM2 was responsible for the OGDR-regulated inflammatory response. TREM2 was induced 6h and 12h after OGDR. Once transfected with TREM2 siRNA fragments, the up-regulated expression of TREM2 was markedly suppressed, whereas untransfected and control siRNA groups showed no changes (Fig. 4a). When TREM2 was silenced, the mRNA levels of the inflammatory mediators TNF-α, IL-1β, and iNOS were increased, whereas IL-10 was decreased (Fig. 4b). We then constructed a TREM2 overexpression plasmid. Transfection of the TREM2-encoding plasmid resulted in marked TREM2 overexpression in microglia (Fig. 4c). This TREM2 overexpression induced a significant suppression of TNF-α, IL-1β, and iNOS, but an increase of IL-10 (Fig. 4d). These data suggested that TREM2 was important for the regulation of the inflammatory response following OGDR and that TREM2 might play a restraining role during the inflammation process.

### Inhibition of TREM2 facilitates inflammation induced by ischemia in MCAO mice

**Fig. 5:**
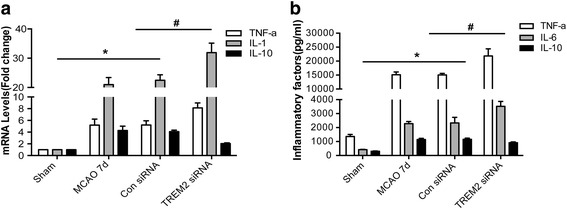
Inhibition of TREM2 facilitates the inflammation induced by ischemia in MCAO mice. MCAO mice were injected with TREM2 siRNA or control siRNA. Tissue homogenates from ischemic cerebral hemisphere (ischemic core and penumbra) of 7 days mice after MCAO under the indicated treatments were subjected to (**a**) quantitative real-time PCR with primers of TNF-α, IL-1β, and IL-10 or (**b**) enzyme-linked immunosorbent assays for TNF-α, IL-1β, and IL-10. Quantified data are shown as mean ± SEM; *n* = 6; *P < 0.05,levels of TNF-α, IL-1β, and IL-10 compared with those in the sham group; ^#^ *P* < 0.05 compared with the control siRNA group injected for 7 days after MCAO

We next investigated whether TREM2 also plays a role during the inflammation process in vivo. MCAO mice were injected with TREM2 siRNA as described in Fig. 3. After silencing the TREM2 gene, the MCAO-induced up-regulated mRNA levels of TNF-α and IL-1β were enhanced, whereas that of IL-10 was inhibited (Fig. 5a). Brain homogenates obtained from ischemic penumbrae were subjected to ELISA to detect the protein levels of the inflammatory factors. The results showed that TNF-α, IL-1β, and IL-10 protein levels were upregulated in mice 7 days after MCAO. The injections of TREM2 siRNA induced a further increase in TNF-α and IL-1β protein expression levels, but a decrease in the IL-10 level (Fig. 5b), consistent with the changes observed in mRNA levels. These data indicated that TREM2 was critical for regulating the inflammatory response in MCAO mice in vivo.

### TREM2 reduces neuronal apoptosis following OGDR in vitro

**Fig. 6:**
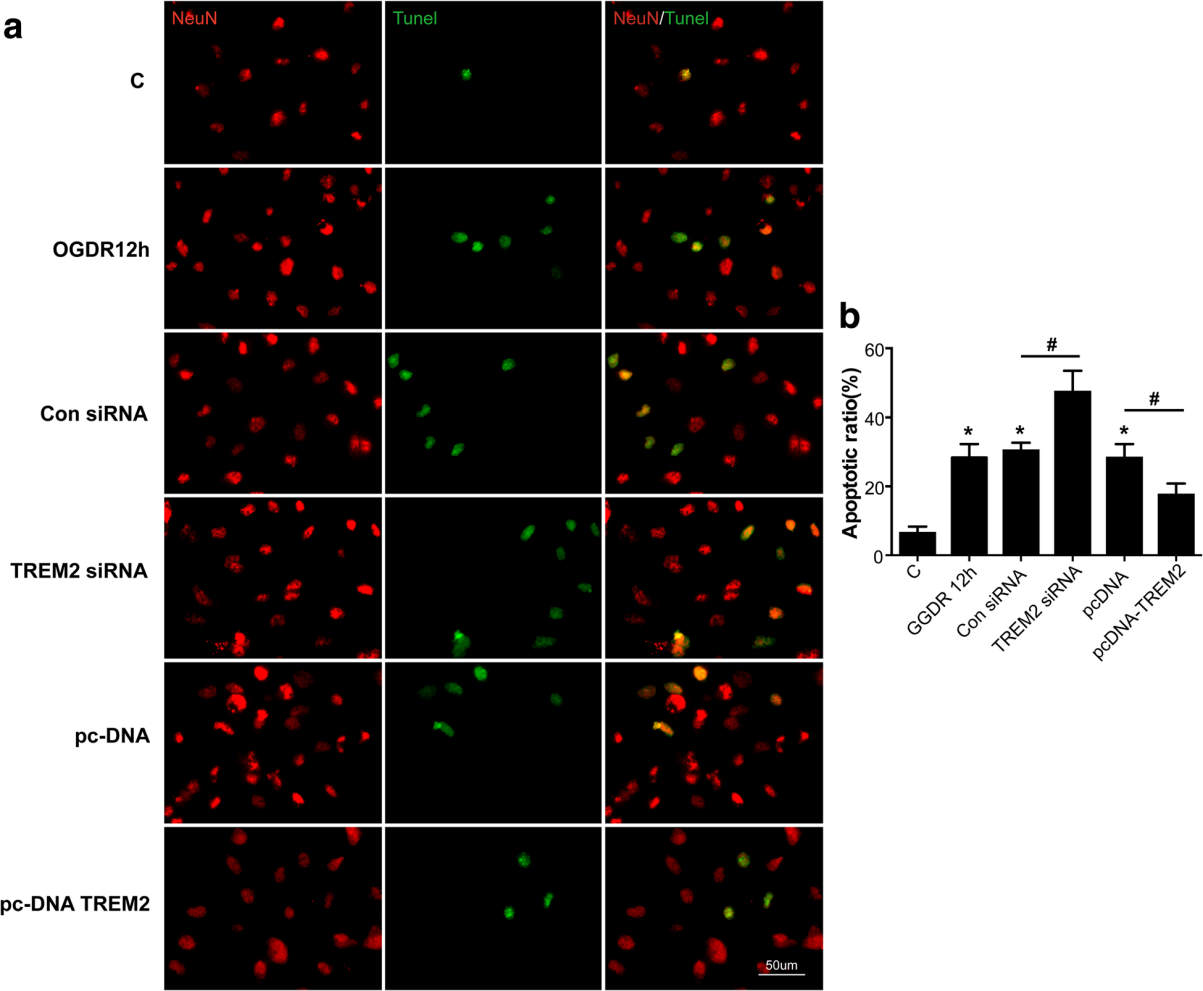
**a** Representative images of cells subjected to OGDR and transfected with either TREM2 siRNA or a TREM2 overexpression plasmid were immunostained with NeuN (*red*) and TUNEL (*green*). Scale bar, 50 μm. **b** The percentage of apoptotic neurons was calculated using image analysis software. Quantified data are shown as mean ± SEM; *n* = 5; *P < 0.05 compared with control group; ^#^ *P* < 0.05 compared with the control siRNA or overexpressing vector groups 12h after OGDR

To investigate the role of TREM2 on neuronal apoptosis, primary hippocampal neurons were co-cultured with microglia in a transwell system. Cells were transfected with TREM2 siRNA or a TREM2-overexpressing vector 12h after OGDR, and apoptotic neuronal cells were detected by double staining with NeuN and TUNEL. OGDR significantly induced neuronal apoptosis. TREM2 knockdown further increased the percentage of apoptotic neuron, whereas TREM2 overexpression rescued neuronal survival (Fig. 6a and b). These data indicated that TREM2 had a protective function against neuronal injury during ischemic stroke.

### Signaling pathways involved in TREM2-mediated neuronal protective function

**Fig. 7:**
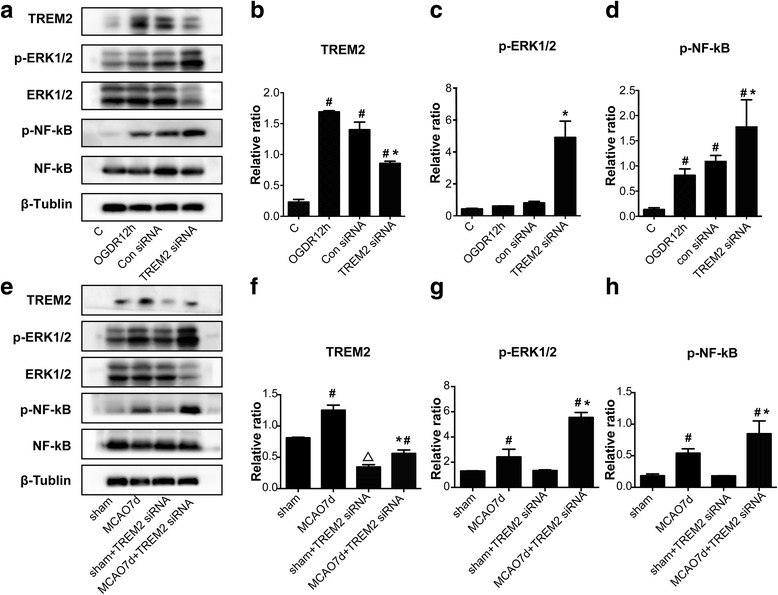
Signaling pathways of TREM2 protection against cerebral injury. **a** TREM2, p-ERK1/2, ERK1/2, p-NF-κB and NF-κB protein expression in cultured primary microglial cells were determined by western blotting 12h after OGDR, with or without transfection TREM2 siRNA or control. **e** These protein expression also in mice (sham, MCAO7d, sham + TREM2 siRNA, MCAO7d + TREM2 siRNA) were determined by western blot. Densitometric analyses of TREM2 versus β-Tublin (**b**,**f**), p-ERK1/2 versus ERK1/2 (**c**,**g**) and p-NF-κB versus NF-κB (**d**,**h**) were show as mean ± SEM; *n* = 5; # *P* < 0.05 compared control (normal) cells or the sham (sham + TREM2 siRNA) groups; *P < 0.05 compared with OGDR 12h cells transfected with control siRNA or MCAO7d mice; Δ*P* < 0.05 compared sham transfected with TREM2 siRNA to sham mice

To explore the possible mechanisms involved in the TREM2-mediated neuronal protective function against cerebral injury, the nuclear factor κB (NF-κB) and ERK transduction pathways in inflammation were examined. The NF-κB and ERK transduction pathways are classical for many proinflammatory factors releasing, including TNF-a,IL-6 and IL-1β which we found regulated by TREM2 [16]. Cultured primary microglia cells were subjected to OGDR for 12h, after the transfection of TREM2 siRNA fragments. OGDR induced the level of TREM2, which can be inhibited by TREM2 siRNA fragments (Fig. 7a and b). We found that the phosphorylation of NF-κB were significantly increased after OGDR treatment, however, TREM2 silence resulted a further augment (Fig. 7a and c). Moreover, the phosphorylation levels of ERK showed no increase in OGDR, but were significantly induced upon TREM2 knockdown (Fig. 7a and d). In addition, we repeated the experiment of testing NF-κB and ERK1/2 phosphorylation in mice 7d following MCAO. Similarly, we found that the phosphorylation of NF-κB and ERK1/2 were significantly increased after MCAO 7d, which were further aggravated in TREM2 knockdown (Fig. 7e, g and h). These data indicated the NF-κB and ERK signaling pathways were responsible in TREM2-mediated neuronal protective function against cerebral injury.

## Discussion

**Fig. 8:**
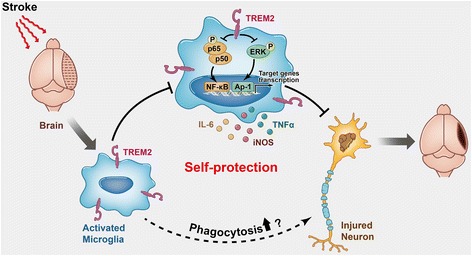
Schematic representation of the role of TREM2 in cerebral ischemia/reperfusion injury

In the current study, we demonstrated that TREM2 expression was enhanced in cultured primary microglia cells after OGDR and in mouse brain after MCAO and that this increased TREM2 mainly occurred in microglia. Both in vivo and in vitro ischemic stroke models induced the production of inflammatory mediators, including TNF-α, IL-1β, iNOS, and IL-10. Genetic knockdown of TREM2 further enhanced the production of TNF-α, IL-1β, and iNOS, but suppressed the production of IL-10. By contrast, the overexpression of TREM2 inhibited the production of TNF-α, IL-1β, and iNOS, but increased the production of IL-10 in cultured primary microglia. The effects on TREM2 knockdown were further confirmed in vivo using MCAO mice. TREM2 silencing in mice exacerbated neuronal apoptosis and neurological dysfunction, whereas TREM2 overexpression in vitro protected neurons against ischemic injury. These data indicated that TREM2 protects against cerebral ischemia/reperfusion injury (Fig. 8).

TREM protein was thought to primarily express in cells of the myeloid lineage [32–34], but TREM has also been detected in other cell types, such as platelets [35] and endothelial cells [36, 37]. Takahashi et al. found that TREM2 is mainly distributed in primary microglia cells [21]. Sessaet al. revealed that TREM2 is located in the Golgi apparatus and can be transferred into cell membranes after stimulation within omycin [38]. Turnbull et al. discovered that IL-4- and IL-13-activated macrophages and macrophages infiltrating from the circulation also express TREM2. Our present data showed that the transcriptional and post-transcriptional levels of TREM2 were both increased and then decreased in microglia after OGDR and in the infarcted marginal zone of MCAO mice. The increased expression of TREM2 was mainly observed in microglia, and not in astrocytes, neurons, and oligodendrocytes. These data are consistent with previous findings [19, 20]. However, we did not determine whether TREM2 was also increased in macrophages infiltrating from the circulation, because innate immune microglia and circulating macrophages were not distinguished. In future studies, using transplants of GFP+ macrophages for tracing, we will be able to distinguish the cells responsible for the increased TREM2 expression.

A genetic mutation of TREM2 causes Nasu-Hakola disease and increases the risk of Alzheimer’s or Parkinson’s diseases. The mechanisms underlying TREM2 mutation-related diseases are unclear, but they may be associated with the disabled function of microglia to phagocytose apoptotic neurons and Aβ proteins. Upon coupling with DAP12, TREM2 transduces extracellular signals by regulating extracellular receptor kinase and the expression of inflammatory mediators, and remodels the cytoskeleton to promote microglial phagocytosis [21]. Intravenously administered TREM2-transduced myeloid precursor cells limit tissue destruction and facilitate repair by clearance of cellular debris during experimental autoimmune encephalomyelitis [22]. Moreover, TREM2 enhances microglial phagocytosis of Aβ_1-42_ and suppresses the Aβ_1-42_-induced pro-inflammatory response. Overexpression of TREM2 in an Alzheimer’s disease transgenic mouse model significantly ameliorates Alzheimer’s disease-related neuropathology, including Aβ deposition, neuroinflammation, and synaptic losses, which is accompanied by improved spatial cognitive functions [17]. Our results in cultured primary microglia subjected to OGDR showed that modulating TREM2 affected the production of inflammatory mediators, including TNF-α, IL-1β, iNOS, and IL-10, consistent with the conclusions fromother studies that TREM2 has anti-inflammatory effects [17, 21]. Additionally, in the transwell co-cultured system, we found that TREM2 was protective against neuronal apoptosis after OGDR. Moreover, injecting TREM2 siRNA fragments into mice previously subjected to MCAO knocked down TREM2, induced the expression of the pro-inflammatory factors TNF-αand IL-1β, and suppressed the expression of the anti-inflammatory factor IL-10, resulting in increased infarct volume, enhanced neuronal apoptosis, and impaired neuronal function. Taken together, these data suggest that TREM2 suppresses the inflammatory response and neuronal apoptosis and promotes brain tissue repair and functional recovery. Besides alleviating the inflammatory response, microglial TREM2 may participate in neuronal protection via removal of dying cells shown by a recent study [19]. Interestingly, it was a contradictory scenario that attenuated inflammatory response in TREM2 knockout mice following stroke [20]. The discrepancy could be explained by that the systemic TREM2 knockout mice were nonspecific representative in brain. Cerebral inflammation is complex and most mediators bear overlapping and pleiotropic functions. Conditional ablation of TREM2 in microglia may present different results at all.

Overall, we found that TREM2 plays a significant role in ischemic brain injury. Agonists of TREM2 would be expected to repair cerebral ischemia/reperfusion injury, making TREM2 an attractive new clinical target for the treatment of ischemic stroke and other cerebrovascular diseases.

## Methods

### Intraluminal middle cerebral artery occlusion model

Adult 6- to 8-week-old male C57BL/6J mice, weighing 20–25g, were housed under diurnal lighting conditions with 12h of light (lights on at 7:00 AM) and allowed access to food and water *ad libitum*. The MCAO model used was previously developed and described [39, 40]. Briefly, after mice were deeply anesthetized with chloral hydrate (200mg/kg, intraperitoneal injection), their fur and skin were disinfected with povidone-iodine (Betadine, Purdue Frederich Company, Norwalk, CT, USA). A midline neck incision was made, and the soft tissues were retracted. Thecommon carotid artery, bifurcation of the internal common carotid artery (ICA), and external common carotid artery (ECA) were carefully dissected. The ECA was temporarily occluded using a 5-0 silk suture. The ICA was clipped using reverse-action tweezers to minimize bleeding. A small hole was cut into the ECA for the insertion of a silicone-coated filament (Beijing Cinontech Co.Ltd, Beijing, China) into the ICA. The suture was tightly tied around the monofilament to prevent bleeding, and the reverse-action tweezers were removed. After 90 min, the suture was removed for reperfusion, and the wound and skin were closed. The body temperature of the mice was maintained between 37.0°C and 37.5°C with a heating pad during surgery. For sham operations, all procedures were identical except that the occluding monofilament was not inserted. There was no significant difference in the average body weight or temperature between groups [41].

### Oxygen-glucose deprivation and reoxygenation model

Primary microglia derived from newborn mice were prepared from mixed glial cultures using the “shaking off” method, as described previously [42]. Briefly, cells were collected and seeded at 1 × 10^6^/mL in uncoated culture flasks with normal culture fluid containing Dulbecco’s modified Eagle medium (DMEM, Gibco, Carlsbad, CA, USA)/F12 supplemented with 10% fetal bovine serum (Gibco, Carlsbad, CA, USA), 1 × 10^5^U/L penicillin (Gibco, Carlsbad, CA, USA), and 1 × 10^5^ U/L streptomycin (Gibco, Carlsbad, CA, USA) sulfate (pH 7.2). After 24h of incubation, the cells were transferred to glucose-free DMEM, and the culture flasks were placed into a sealed tank with a persistent low flow (1.5 L/min) of 94% N_2_, 5% CO_2_, and 1% O_2_ for 4h. After 4h of oxygen-glucose deprivation, reoxygenation was achieved by changing the medium to normal culture fluid and by exposing the cells to ambient air. Real-time (RT)-PCR, western blot, and immunofluorescence assays were performed on the cells 0, 6, 12, 24, 48, and 72h after OGDR. The control group was cultured in normal culture medium, with cells exposed to ambient air.

### Microglia TREM2 siRNA transfection

Normal culture medium was used, and cells were exposed to ambient air until transfection. The TREM2 siRNA mixtures (TREM2-siRNA1, 5′-GAGGGUGUCAUGUACUUAUTT-3′; TREM2-siRNA2,5′-CCUCUAGAUGACCAAGAUTT-3′;TREM2-siRNA3,5′-GGAAUCAAGAGACCUCCUUTT-3′) or control siRNA (5′-UUCUCCGAACGUGUCACGUTT-3′) was transfected into primary microglia cells for 36h using siRNA or RNAi-Mate (a mixture of control siRNA and RNAi-Mate, GenePharma, Shanghai, China) using the protocol provided by the manufacturer. Following transfection, the cells were exposed to OGDR as described above.

### TREM2 siRNA transfer into mouse brain

As described previously [26, 27], mice were placed 10 min after successful MCAO model establishment into a stereotactic frame (Huaibei Zhenghua Biologic Apparatus Facilities Limited Company, Anhui, China). Each mouse received 3 μL of TREM2 siRNA or the mixture of control siRNA (1.8μLof control siRNA + 0.8 μL of RNAi-Mate + 0.4 μL of ddH20, mixed at 20°C for 20 min), which was prepared by GenePharma (Shanghai, China). The liquid was slowly injected into the lateral ventricles at a rate of 0.2 μL/min via a mini-pump (RWD, Shenzhen, China). For successive intracerebroventricular injection (7 days), the indwelling catheters (RWD, Shenzhen, China) were fixed to the skull with dental cement.

### Plasmids

The control plasmid pLV(ExSi)-Puro-CMV-eGFP(pcDNA) and the TREM2-encoding plasmid (pLV(Exp)-Puro-CMV-mTREM2-eGFP, pcDNA-TREM2) were constructed by Cyagen Biosciences Inc. (Guangzhou, China). All plasmid sequences were confirmed by gene sequencing.

### TUNEL assay

Apoptotic neuronal cells were detected via double staining with NeuN (1:500, Millipore, USA) and TUNEL (FragEL DNA Fragmentation Detection Kit, Fluorescent-TdT Enzyme; Merck-Millipore, Germany). The mice used in the ischemic stroke model were killed, and brains were sliced into coronal sections 30 μm thick. The ratio of apoptotic to live neuronal cells was calculated.

### Immunofluorescence

The immunostaining procedure was performed as previously described [43]. At the indicated times after MCAO, the brains were removed quickly and post-fixed in 4% paraformaldehyde overnight. The brains were then dehydrated in a gradient sucrose solution (10%, 20%, and 30%) at 4°C. Serial sections of the mouse brain (10 μm thick) were cut using a cryostat. Microscope slides with mounted brain slices were stored temporarily in cryoprotectant solution at 30°C until use for morphological staining. After rinsing with 0.3% Triton X-100 for 30 min at 37°C, the sections were blocked in a solution containing 5% normal goat serum for 1h, and then washed several times. The sections were incubated with the following primary antibodies at 4°C overnight: polyclonal anti-Iba1 (1:400), monoclonal anti-NeuN (1:300), anti-GFAP (1:1000), and rabbit anti-BMP (1:500). The primary antibodies were detected by Alexa488- and Alexa594-conjugated secondary antibodies (1:1000) at room temperature for 1h. The sections were coverslipped with glycerol, and the immunofluorescence was detected using an Olympus BX5 microscope. The percentage of apoptotic neurons was determined for each group of each repeated experiment (200× magnification).

For immunocytochemistry, the cells were washed twice with PBS, fixed with 4% paraformaldehyde for 20 min, and then blocked with 5% goat serum albumin for 1h. The cells were then incubated with antibodies at 4°C overnight, washed 3 times with PBS, incubated with rat anti-rabbit Cy3-conjugated secondary antibodies (1:1000) for 1h in a 37°C thermostat-controlled incubator, and finally coverslipped with an anti-fluorescence-quenching reagent (Boshide, China). After staining the cell nuclei with 4',6-diamidino-2-phenylindole (DAPI), the fluorescent images were detected and analyzed as described above. For each repeated experiment, the cells were counted in 5 random fields (200× magnification).

For terminal deoxynucleotidyl transferase dUTP nick end labeling (TUNEL) assays, samples obtained following OGDR or MCAO were doublestained using a NeuN and TUNEL kit, according to the manufacturer’s instructions. The fluorescence imaging procedure was performed and analyzed as described above.

### Real-time PCR

Total RNA was isolated from primary microglia cells or mouse brain tissue in the fringe area of the infarct using the RNeasy Plus Mini Kit (TaKaRa), according to the manufacturer’s instructions. After reverse transcription, quantitative real-time PCR was performed using primers specific for the genes encoding TREM2 and the inflammatory mediators IL-1β, IL-10, inducible nitric oxide synthase (iNOS), and TNF-α. Fast thermal cycling was performed using a real-time PCR system (Roche LightCycler 480) under the following conditions: pre-denaturation at 95°C for 10 min, followed by 40 cycles at 95°C for 15s, 57°C for 30s, and 72°C for 30s. Semi-quantitative PCR experiments were also performed using primers that specifically amplified the C-terminal region of the full-length transcript encoding TREM2. The results were expressed as the relative mRNA expression of the threshold cycle value, and were normalized by parallel amplification of the endogenous control GAPDH. The relative mRNA expression level in the control group (target mRNA/GAPDH value) was set to 100%, and the mRNA values in other groups were converted to fold changes after comparison with the control group.

### Western blotting

Cultured cells and brain tissues were lysed in extraction buffer. Different samples with an equal amount of protein were separated on sodium dodecyl sulfate polyacrylamide gels, transferred to polyvinylidene fluoride membranes, and blocked with 5% bovine serum albumin. Membranes were incubated overnight with primary antibodies against TREM2 (Abcam, USA) and GAPDH (Abcam, USA), and then washed again and incubated with horseradish peroxidase-coupled secondary antibody. Protein bands were detected with a chemiluminescent horseradish peroxidase substrate (CWBio, China). The relative densities of bands were analyzed with a gel imaging analysis system (Genetics Inc., USA).

### Enzyme-linked immunosorbent assay (ELISA)

Brain homogenates obtained from ischemic penumbra and primary microglial lysates were prepared with cold PBS. TNF-α, IL-1β, and IL-10 levels in homogenate and cell supernatants were estimated using a mouse ELISA kit (DAKEWE, Shenzhen, China) according to the manufacturer’s instructions.

### 2,3,5-triphenyl-tetrazolium-chloride (TTC) staining

Brains were isolated and cut into 2mm-thick coronal sections. The 2mm sections were incubated in a 2% TTC solution for 30 min at 37°C in the dark. The staining process was stopped by 4% paraformaldehyde in PBS. The stained sections were scanned after 12h, and the infarct volume was determined by image analysis and expressed as the percentage of the whole cerebral tissue.

### Neurological function evaluation

Signs of neurological deficits allow for the evaluation of the success of the MCAO model immediately after reperfusion and later for the estimation of the degree of severity of the injury. Neurological deficits are acceptably scored using the Modified Garcia Score [44], an 18-point sensorimotor assessment system consisting of 6 tests, with scores of 0–3 for each test (maximum scores = 18). These 6 tests include: (i) spontaneous activity, (ii) side stroking, (iii) vibrissae touching, (iv)limb symmetry, (v)climbing, and (vi) forelimb walking.

### Data analysis

All mice were randomly assigned to the experimental and control groups. All experiments were repeated at least 5 times, and the results are presented as the mean ± SEM. The differences were analyzed for statistical significance using Student’s *t*-tests for 2 groups and one-way ANOVA with Bonferroni corrections for multiple group comparisons. Values of *P* less than 0.05 were considered statistically significant.

## Additional file

Additional file 1: Figure 1. TREM2 colocalizes with Iba1^+^microglia, but not with NeuN^+^ neurons, MBP^+^myelin, or GFAP^+^ astrocytes in mice subjected to normal mouse brain. Scale bar, 50 μm. (TIF 5197 kb)

## Abbreviations

CNS: The Central nervous system
IL-10: Interleukin-10
IL-1β: Interleukin-1β
IL-6: Interleukin-6
iNOS: Inducible nitric oxide synthase
MCAO: The middle cerebral artery occlusion model
OGDR: Oxygen-glucose deprivation and re-oxygenation
TNF-α: Tumor necrosis factor
TREM2: Triggering receptor expressed on myeloid cells 2
