# Supplementary figures and images for: TREM2 protects against cerebral ischemia/reperfusion injury
Source: Mol Brain. 2017 Jun 7;10:20. doi: 10.1186/s13041-017-0296-9 (PMC5461720; doi:10.1186/s13041-017-0296-9)

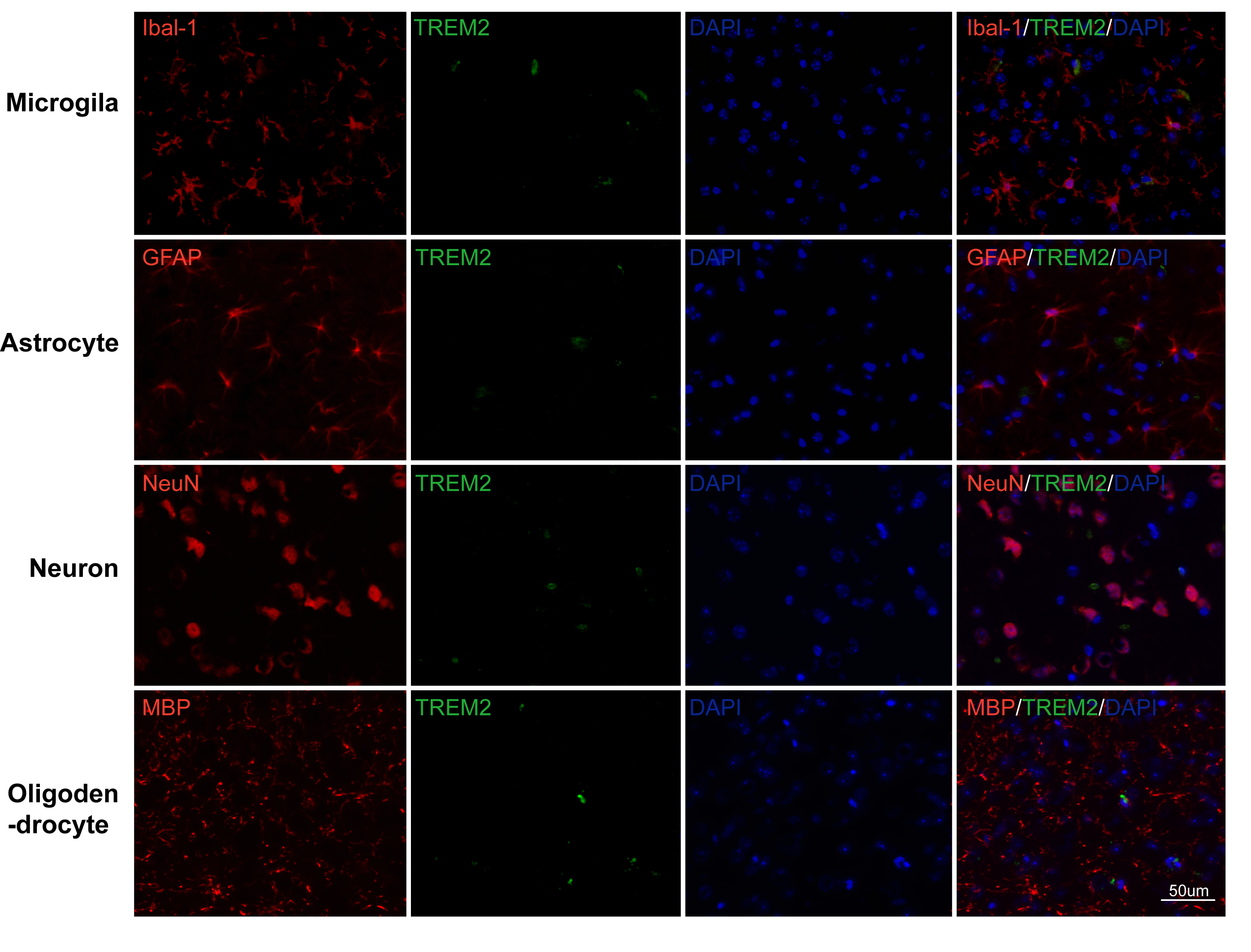

Supplement: Additional file 1: Figure 1. — TREM2 colocalizes with Iba1+microglia, but not with NeuN+ neurons, MBP+myelin, or GFAP+ astrocytes in mice subjected to normal mouse brain. Scale bar, 50 μm. (TIF 5197 kb) [file 13041_2017_296_MOESM1_ESM.tif]
